# *om92* , a *glp-1* enhancer mutation, is an allele of *ekl-1*

**DOI:** 10.17912/micropub.biology.000698

**Published:** 2022-11-30

**Authors:** Samantha A. Stein, Olivia F. Zucaro, Harold E. Smith, Kevin F. O'Connell, Jill M. Spoerke, Eleanor M. Maine, James L. Lissemore

**Affiliations:** 1 Biology Dept., John Carroll University, University Heights, OH USA; 2 National Institute of Diabetes and Digestive and Kidney Diseases, National Institutes of Health, Bethesda, MD USA; 3 Laboratory of Biochemistry and Genetics, National Institutes of Diabetes and Digestive and Kidney Diseases, NIH, Bethesda, MD USA; 4 Biology Dept., Syracuse University, Syracuse, NY USA

## Abstract

Germline stem cell proliferation in *C. elegans* requires activation of the GLP-1/Notch receptor, which is located on the germline plasma membrane and encoded by the *glp-1* gene. We previously identified several genes whose products directly or indirectly promote activity of the GLP-1 signaling pathway by finding mutations that enhance the germline phenotype of a *glp-1(ts)* allele, *glp-1(bn18)* . Here, we report phenotypic and molecular analysis of a new *ekl-1* allele, *ekl-1(om92)* , that enhances the *glp-1(bn18)* phenotype. *ekl-1(om92)* is a 244 bp deletion predicted to generate a frameshift and premature termination codon, yielding a severely truncated protein, suggesting it is a null allele.

**Figure 1. glp-1(bn18) enhancer allele om92 disrupts germline function and is likely a null allele of the ekl-1 gene:**
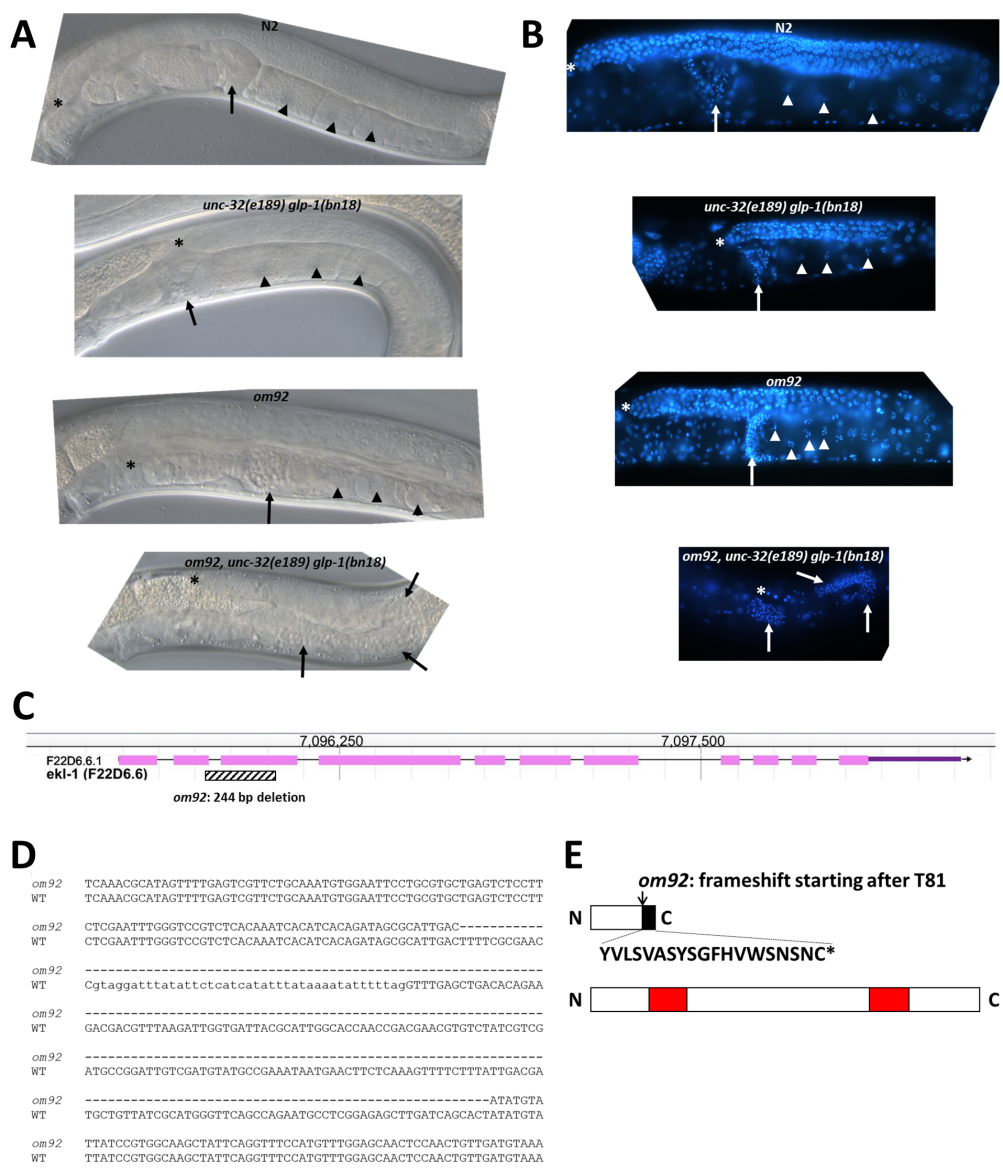
(A) DIC images of representative germlines from young adult hermaphrodites of the indicated strains raised at 20 °C. Asterisks indicate the distal tip cell. Arrows indicate location of sperm. Arrowheads indicate location of oocytes. (B) Fluorescence images of representative DAPI-stained germlines from young adult hermaphrodites of the indicated strains raised at 20 °C. Marking symbols are the same as in (A). (C) Screenshot of *ekl-1* gene location on LG I and gene organization from Wormbase (WS285, https://wormbase.org) indicating location and length of the *ekl-1(om92)* deletion. (D) Partial sequence alignment of *ekl-1(om92)* and *ekl-1(+)* genomic sequence indicating precise extent and location of the *ekl-1(om92)* deletion. Exon sequences are uppercase while intron sequences are lowercase. WT, wildtype. (E) Schematic diagram of predicted proteins encoded by *ekl-1(om92)* (above) and *ekl-1(+)* (below). Red boxes in the predicted wildtype EKL-1 protein indicate locations of Tudor domains according to UniProt (Release 2022_03, https://www.uniprot.org/). The black box in the predicted mutant EKL-1 protein indicates the extent of the amino acid sequence changes caused by the *ekl-1(om92)* deletion and resulting frameshift. The C-terminal amino acid sequence resulting from the frameshift (after residue T81) is shown. The predicted length of the mutant EKL-1 protein is 100 amino acids, and the wildtype protein is 606 amino acids.

## Description

We have reported the identification of numerous genes that promote GLP-1/Notch signaling in the *C. elegans* germline through genetic analysis of EMS- and UV/TMP-induced mutations in a sensitized genetic background. Specifically, we have found a series of *ego* ( e nhancer of *glp- o ne* ) alleles that genetically enhance the reduced germline proliferation observed in a temperature-sensitive allele of *glp-1,* *glp-1(bn18)* (Qiao, *et al* ., 1995; Smardon, *et al.,* 2000; Liu and Maine, 2007; She, *et al* ., 2009; Lissemore, *et al.,* 2018). Molecular characterization of these *ego* alleles revealed genes that directly or indirectly promote GLP-1/Notch signaling, including the transcriptional regulator *lag-1* , cell cycle regulatory genes *cye-1* and *cdk-2* , and several genes encoding small RNA machinery components ( *drh-3, ekl-1, ego-1* ), among others (Qiao, *et al* ., 1995; Smardon, *et al* ., 2000; She, *et al* ., 2009; Fox, *et al* ., 2011). We present here molecular and phenotypic analysis of another allele from our previously reported UV/TMP screen for *ego* alleles, *om92.*

Wildtype young adult hermaphrodites have active germline proliferation (i.e., a progenitor zone) in the distal gonad arm (Figure 1A and 1B). As germline cells move proximally, they enter meiosis and in adults undergo oogenesis; oocytes mature in the proximal region prior to fertilization in the spermatheca. *unc-32(e189) glp-1 (bn18)* young adult hermaphrodites at 20 °C have reduced GLP-1 signaling, leading to a reduced progenitor zone compared to wildtype (Figure 1A and 1B). *unc-32(e189)* is tightly linked to *glp-1* and is used here as a marker for the presence of *glp-1(bn18).* The overall organization of the *glp-1(bn18)* germline at 20 °C is the same as in wildtype although the size is somewhat smaller. Young adult hermaphrodites homozygous for *om92* , a UV/TMP-induced mutation, are sterile but have a substantial germline (Figure 1A and 1B). The distal region contains a progenitor zone, and more proximal germ cells undergo meiosis, forming sperm and sometimes oocytes; when present, oocytes are small and morphologically abnormal, as in the examples shown here. In addition, some oocytes appear to be aneuploid or to have a chromosome-pairing defect (i.e., > 6 DAPI-bright foci at diakinesis). Sperm appear normal and are located in the spermatheca. *om92; unc-32(e189) glp-1(bn18)* young adult hermaphrodites have abnormal germlines without distal mitotic cells or meiotic cells, and the size of the germline is correspondingly much smaller than wildtype (Figure 1A and 1B). Oocytes are absent, and sperm are visible scattered throughout the gonad arm. Based on this phenotype, it appears the germline progenitor zone was not maintained during larval development; instead, all germ cells entered meiosis and underwent spermatogenesis prior to young adult stage. Because *om92; unc-32(e189) glp-1(bn18)* germlines do not retain germline stem cells whereas *om92* and *glp-1(bn18)* single mutant homozygotes do, *om92* meets the criterion for an *ego* mutation.

To determine the molecular identity of *om92* , we used the one-step whole genome sequencing and SNP mapping method (Doitsidou, et al., 2010) to delimit the mutation to the region between 6.5-7.5 Mbp on chromosome I. Variant calling (Smith, et al., 2016) revealed a single novel candidate mutation within that interval, a 244 bp deletion in the *ekl-1* gene, that was confirmed by Sanger sequencing of PCR amplicons from *om92* homozygotes (Figure 1C and 1D). *ekl-1* encodes a Tudor domain protein with multiple known germline functions, including chromosome segregation and small RNA biogenesis and stability (Rocheleau, *et al.* , 2008; Claycomb, *et al.* , 2009; Thivierge, *et al.* , 2011). She, *et* *al.* (2009) previously showed, using RNAi and mutational analysis, that loss of *ekl-1* function enhances *glp-1(bn18).* The *om92* deletion removes parts of exons 2 and 3 along with all of intron 2 and is predicted to generate a frameshift downstream of codon 81 (Figure 1E, black box in upper figure). Full-length EKL-1 is predicted to include 606 amino acids. In contrast, the truncated EKL-1 protein is predicted to encode only 100 amino acids and lack both Tudor domains, which have been shown in other organisms to bind to methylated amino acid residues in histones and other proteins (Lorton and Shechter, 2019), hence it is very likely to be non-functional. Thus, *ekl-1(om92)* should be considered a null mutation.

The phenotype of *ekl-1(om92)* homozygotes generally resembles the phenotypes reported for *ekl-1(om56)* and *ekl-1(om83)* (She, et al., 2009). Specifically, we note the presence of small, abnormal oocytes in a subset of mutants. In addition, She, et al., 2009 noted incompletely penetrant chromosomal pairing defects in the oocytes of *ekl-1(om53)* and *ekl-1(om86)* , similar to what we observe here for *ekl-1(om92)* . (These similarities were not noted in the preliminary phenotypic analysis conducted at the time *ekl-1(om92)* was isolated, and therefore She et al. did not analyze *ekl-1(om92).* ) We do not, however, observe variable sperm morphology in *ekl-1(om92)* as She, et al., 2009, reported for *ekl-1(om56)* and *ekl-1(om83)* . We do not have a definitive explanation for the observed phenotypic differences, but they might be attributed to the substantial differences in the predicted protein products from the different alleles. The *ekl-1(om92)* predicted protein product contains only 100 amino acids and lacks both Tudor domains. In contrast, *ekl-1(om83)* is a deletion mutation resulting in a predicted protein product containing 314 amino acids while *ekl-1(om56)* is a nonsense mutation resulting in a predicted protein product with 318 amino acids. Both proteins would contain the first Tudor domain which might result in these proteins having some residual function that the protein encoded by *ekl-1(om92)* lacks.

## Methods

Nematode strains and worm maintenance :

Worms were maintained using standard methods.

Microscopy :

DIC and DAPI microscopy were carried out essentially as described in Lissemore, *et al.,* 2018.

SNP mapping/whole genome sequencing (WGS) :

Combined SNP mapping and WGS were carried out essentially as described (Smith, *et al* ., 2016) using genomic DNA isolated from *om92; unc-32(e189) glp-1(bn18* ) sterile homozygous worms.

PCR and Sanger DNA sequencing :

Single worm PCR was carried out on *ekl-1(om92)* homozygous sterile worms and N2 worms using standard methods. Two different pairs of primers flanking the putative deletion were tested, eklA/eklB and eklC/eklD, and each pair gave the same results. Sanger DNA sequencing was carried out by Eton Bioscience, Inc. (San Diego, CA); primers used for PCR were also used for sequencing amplicons.

## Reagents

Primers

**Table:** 

**PRIMER NAME**	**SEQUENCE (5’-->3’)**
eklA	ATGATTGCGGTTGGACTCCG
eklB	TGGTCACAGATAATGGGCGA
eklC	CCGAATCCGGAAGAACCGAT
eklD	GGAGAGAACAACTTCCACACG

Strains

**Table:** 

**STRAIN**	**GENOTYPE**	**AVAILABLE FROM**
N2	*Caenorhabditis elegans*	CGC
JL50	*ekl-1(om92)/+*	Lissemore Lab
JL51	*ekl-1(om92)/+; unc-32(e189) glp-1(bn18)*	Lissemore Lab
JL52	*ekl-1(om92) I/hT2[bli-4(e937) let-?(q782) qIs48]; unc-32(e189) glp-1(bn18)/hT2[bli-4(e937) let-?(q782) qIs48]*	Lissemore Lab
